# Suppressed inflammation in obese children induced by a high-fiber diet is associated with the attenuation of gut microbial virulence factor genes

**DOI:** 10.1080/21505594.2021.1948252

**Published:** 2021-07-08

**Authors:** Hui Li, Guojun Wu, Liping Zhao, Menghui Zhang

**Affiliations:** a State Key Laboratory of Microbial Metabolism and Joint International Research Laboratory of Metabolic and Developmental Sciences, School of Life Sciences and Biotechnology, Shanghai Jiao Tong University, Shanghai, P. R. China; b Ministry of Education Key Laboratory for Systems Biomedicine, Shanghai Centre for Systems Biomedicine, Shanghai Jiao Tong University, Shanghai, China; c Department of Biochemistry and Microbiology and New Jersey Institute for Food, Nutrition and Health, School of Environmental and Biological Sciences, Rutgers University, NJ, USA

**Keywords:** Virulence factor, obesity, inflammation, high-fiber diet, prader-willi syndrome, gut microbiota, metagenomic

## Abstract

In our previous study, a gut microbiota-targeted dietary intervention with a high-fiber diet improved the immune status of both genetically obese (Prader-Willi Syndrome, PWS) and simple obese (SO) children. However, PWS children had higher inflammation levels than SO children throughout the trial, the gut microbiota of the two cohorts was similar. As some virulence factors (VFs) produced by the gut microbiota play a role in triggering host inflammation, this study compared the characteristics and changes of gut microbial VF genes of the two cohorts before and after the intervention using a fecal metagenomic dataset. We found that in both cohorts, the high-fiber diet reduced the abundance of VF, and particularly pathogen-specific, genes. The composition of VF genes was also modulated, especially for offensive and defensive VF genes. Furthermore, genes belonging to invasion, T3SS (type III secretion system), and adherence classes were suppressed. Co-occurrence network analysis detected VF gene clusters closely related to host inflammation in each cohort. Though these cohort-specific clusters varied in VF gene combinations and cascade reactions affecting inflammation, they mainly contained VFs belonging to iron uptake, T3SS, and invasion classes. The PWS group had a lower abundance of VF genes before the trial, which suggested that other factors could also be responsible for the increased inflammation in this cohort. This study provides insight into the modulation of VF gene structure in the gut microbiota by a high-fiber diet, with respect to reduced inflammation in obese children, and differences in VF genes between these two cohorts.

## Introduction

Trillions of bacteria reside in the human gut and interact with the host. Among them, pathogenic bacteria produce a variety of molecules, called virulence factors (VFs), which assist these pathogens in causing diseases. Higher levels of VF genes have been found in patients with colorectal carcinoma [1], atherosclerotic cardiovascular disease [2], type 2 diabetes (T2D) [3], or alcohol dependence syndrome [4]. In particular, VFs are considered to have important roles in triggering inflammation by interacting with the host immune system [5–7]. Clinical studies have reported that VFs, such as toxins, peptidoglycans, exoenzymes, lipoproteins and glycolipids, can initiate host inflammatory processes [5,8]. Earlier, research on pathogens was mainly restricted to a single gut pathogen and lacked comprehensive analysis of the interactions among different gut pathogens. In recent years, studies on clinically isolated pathogens and the rapid development of sequencing technology have greatly enhanced our knowledge of VFs and the genes encoding them; this has resulted in the development of VF-specific databases, which allow simultaneous analysis of the composition and functions of various pathogens that exist in the gut and the complex interactions among them using metagenomics.

VFs are involved in multifaceted pathogenic mechanisms, and presently, different VF databases have different criteria for their classification [9]. Nevertheless, the VFs and the genes that encode them are generally categorized according to the type of pathogenesis in which they are involved. For example, the Virulence Factor Database (VFDB) is an integrated and comprehensive online resource of VFs, which classifies VFs into four categories according to their verified functions, namely, offensive, defensive, nonspecific, and regulative [10,11]. The offensive category is essential for efficient cellular invasion; it contains proteins that are involved in motility, attachment to host cells, and protection against the host defense system [12,13], toxins that disrupt host cellular function and degrade host tissues [14], and secretion systems that deliver effector proteins across the outer membrane and manipulate various host cell processes [15,16]. The defensive category contributes to host immune evasion and growth for bacteria under stress conditions [17,18]. The nonspecific category consists of VFs involved in basic survival and growth of bacteria [19], such as iron or magnesium uptake. The regulative category mainly participates in the control, regulation, and mediation of other VFs. In fact, many VF genes have been discovered not only in pathogens but also in commensal bacteria [20,21]. Therefore, another classification system classifies VF genes into pathogen-specific and common VF genes, wherein the latter exist in both pathogenic and commensal bacteria [22].

Prader-Willi Syndrome (PWS) is an inherited disease that is caused by a genetic deficit in the human chromosomal region 15q11.2-q13.2 and usually leads to morbid obesity [23]. In our previous study, gut microbiota-targeted dietary intervention in the form of a high-fiber diet was performed for two groups including simple obese (SO) and PWS affected children [24]. We observed that a high-fiber diet could obviously reduce body weight, serum antigen load, and inflammation level, improve glucose and lipid homeostasis status, and alter the overall gut microbial structure in both groups. Although there was no considerable difference between the two groups in the microbiota structure before or after intervention, the measured six inflammation indexes in the PWS group were consistently higher than those in the SO group [24]. These indexes included C-reactive protein (CRP), serum amyloid A protein (SAA), α 1 acid glycoprotein (AGP), white blood cell count (WBC), interleukin 6 (IL-6), and adiponectin. CRP, SAA, and AGP are acute phase proteins (APPs), which are used to assess the response of the innate immune system to inflammation. APPs are usually produced in the liver to enhance protection against invading pathogens, limit tissue damage, and promote a rapid return to homeostasis [8,25,26]. WBC, IL-6, and adiponectin reflect the recruitment of immune cells and the acquisition of immunity.

Some reports have indicated that obesity, insulin resistance, and inflammation are closely correlated with gut pathogens and their VFs [27,28]. It has also been reported that inflammation is linked to obesity and metabolic syndromes, such as insulin resistance, atherosclerosis, and dyslipidemia, among others [29–31]. Therefore, how the gut microbial VF genes affect inflammation in obese children needs to be addressed, in addition to the roles that these genes play in improvement in inflammation in obese children administered a high-fiber diet. To answer these questions, we performed a systematic comparative analysis of VF genes between PWS- and SO-group children during the dietary intervention in this study. Based on metagenomic datasets obtained from the fecal samples of both groups, we investigated the effects of dietary intervention on gut VF genes, compared and identified key VF genes, and clustered candidate VF genes relevant to the inflammation indexes in obese children (WBC, IL-6, CRP, SAA, AGP, and adiponectin).

## Materials and methods

### Ethics statement

The clinical trial was approved by the ethics committee of the School of Life Sciences and Biotechnology, Shanghai Jiao Tong University (No. 2012–016), and registered at the Chinese Clinical Trial Registry (No. ChiCTR-ONC-12,002,646) [24]. The study was performed in accordance with the approved guidelines. Written informed consent was obtained from the guardians of all participants, and the study protocol conformed to the ethical guidelines of the Declaration of Helsinki (2008).

### Clinical trial

The clinical trial was performed as described in our previous study [24]. Briefly, SO (n = 19)- and PWS (n = 17)-group children were screened for the clinical trial and completed a high-fiber dietary intervention at the Guangdong Women and Children hospital for 30 and 90 days, respectively. The diet, rich in whole grains, traditional Chinese medicinal foods, and prebiotics (WTP diet) [32], was administered according to the dietician’s advice. All obese children were allowed to consume enough of a pre-cooked mixture to satisfy hunger, including whole grains, adlay, oat, buckwheat, white bean, yellow corn, red bean, soybean, yam, peanut, lotus seed, and wolfberry. Additionally, a powder preparation for infusion containing bitter melon, oligosaccharides, and soluble prebiotics, including Fibersol-2, fructo-oligosaccharide, and oligoisomaltose (50 g), was supplied to volunteers every day. The dietary intake was balanced according to the age standard nutritional requirement provided by the Chinese Dietary Reference Intakes (DRIs) recommended by the Chinese Nutrition Society (CNS, 2012).

### Data collection and pre-processing

The physiological indexes and fecal samples of all participants were collected at pre-defined time points (SO: day 0 and 30; PWS: day 0, 30, 60, and 90) [24]. The six inflammation indexes used in this study were CRP, SAA, AGP, WBC, IL-6, and adiponectin. After collection, the feces were immediately frozen on dry ice and stored at −80°C. DNA of the fecal samples was extracted according to a standard protocol (including centrifugation, resuspension, transfer, buffering, incubation, washing, etc.) [33]. DNA was purified with the QIAamp DNA mini kit (51,304, QIAGEN, Germany) according to the manufacturer’s instructions. Metagenomic sequencing of the purified DNA was performed on an Illumina Hiseq 2000 platform at Shanghai Biotechnology Co., Ltd.

The PWS-group children were distinguished from the SO children through molecular genetic diagnosis [34,35]. Briefly, the DNA was isolated from the peripheral blood of all obese children and their parents, and the extracted DNA was treated with sulfite using the CpGenome DNA modification kit. Then, the amplicons were obtained from paternal and maternal chromosomal DNA using methylation-specific primers in the CpG island of the *SNRPN* gene in region 15q11.2-q13.2. Finally, electrophoresis was performed on the amplification products. Individuals with missed paternal electrophoretic band were diagnosed as PWS patients.

The original sequencing data can be accessed at the NCBI SRA database with accession number SRP045211 [24]. The data pre-processing was performed as described in our previous study [24]. Briefly, Flexbar [36] was used to trim the adapters from the reads, and Prinseq [37] was employed for initial quality control of the reads. The reads that aligned to the human genome (*Homo sapiens*, UCSC hg19) with Bowtie2 [38] were removed. As a result, an average of 84.6 ± 21.2 million (mean ± s.d.) high-quality reads per sample were obtained and used in this study (see Supplemental Table S1 for details).

### Bioinformatics analysis

To ensure analytical preciseness and avoid multiple alignments, we selected the core dataset of VFDB (http://www.mgc.ac.cn/VFs/download.htm), which is a non-redundant database and only includes experimentally verified VF genes, as the VF reference database. This dataset contains 2,598 VF genes (by 21 March 2017) and provides the nucleotide sequence, encoded protein, bacteria, classification, function, GenBank accession number, and characteristics, among others, of VF genes based on original literature or reviews. For each sample, the obtained high-quality sequences were aligned to the reference database using Burrows-Wheeler Aligner version 0.7.15 with default settings [39]. To control data variation, we discarded the inaccurate and low-quality alignments as follows: 1) an alignment score < 30; 2) paired-end reads that aligned to none of the VF genes or aligned to different VF genes; 3) alignment bases fewer than 45. Then, for any sample S, the relative abundance of the *i*th VF gene $A_{i}$was calculated as follows:

(1) $$A_{i}=\frac{N_{i}}{L_{i}\times T_{s}\;}$$

where $N_{i}$ refers to the number of sequences mapped to VF gene i, $L_{i}$ is the length of VF gene in per kilobase of sequences, and $T_{s}$ is the total number of sequences per million sequences of sample . To make the conclusions more generalizable, we set the criteria for detecting VF genes, and only the genes that appeared in more than 20% of PWS or SO cohort samples of were considered detected VF genes and kept for downstream analysis.

Two methods of VF gene classification were adopted in this study. Pathogen-specific and common genes were classified according to the categories supplied by Nui et al [22]. Genes that only existed in pathogens were pathogen-specific genes, whereas those that occurred in both pathogens and commensal bacteria were common genes. In addition, this study further refined the classification of the detected VF genes based on the annotation provided by VFDB, to ensure that each class was both detailed and general. In total, 17 classifications are obtained, including adherence, iron uptake, endotoxin, T3SS (type III secretion system), antiphagocytosis, invasion, exoenzyme, toxin, T2SS (type II secretion system), protease, stress protein, T6SS (type VI secretion system), biofilm formation, efflux pump, immune evasion, manganese uptake, and T7SS (type VII secretion system).

### Statistical analysis

Random Forest (RF) analysis was used to screen featured VF genes using the “sklearn” package (Version 0.18.2) in python (Version 2.7.13). The optimal RF model was determined through the “GridSearchCV” function. Only VF genes with a feature score > 0 and a fold-change ≥ 8 were kept as featured differential VF genes for downstream analysis.

The significance of differences in VF genes, classes, or inflammation indexes between before and after dietary intervention in the same cohort were judged with a two-tailed Wilcoxon matched-pairs signed rank test using R software (version 3.5.0), whereas differences in VF genes, classes, or inflammation indexes between the two cohorts were assessed with the Wilcoxon unpaired rank test. The Benjamini-Hochberg post hoc procedure was applied for the adjustment of P values. Pairwise comparisons of VF gene abundance between two groups or one group at different intervention times were conducted by the ANOVA permutation test using “Euclidean” distance matrices (permutation = 999). Permutational multivariate analysis of variance (PERMANOVA) in the “vegan” library of R software was applied for differences among multiple groups (permutation = 9,999). Heatmaps and radar charts of VF genes at different intervention times were generated in R using packages “pheatmap” (version 1.0.8) and “ggradar” (version 0.1), respectively. Unsupervised hierarchical clustering of the two cohorts at different intervention time points was performed by “Euclidean” and “Complete” methods based on the average abundance of the VF genes. Spearman’s correlation coefficients between VF genes and inflammation indexes were calculated in R. The co-occurrence networks were constructed using Cytoscape software (version 3.3.0) [40] based on Spearman’s correlation coefficients > 0.6 for VF–VF gene comparisons or > 0.3 for VF gene–clinical index comparisons. The Cytoscape plugin “MCODE” was used for the auxiliary detection of the clustered VF genes closely related to inflammation indexes.

## Results

### Global shift in the gut microbial VF genes in PWS- and SO-group children during the dietary intervention

The dietary intervention caused impressive changes in the gut microbiota VF genes in both PWS and SO groups. In total, 408 VF genes were detected in the gut microbiota of the PWS- or SO-group children throughout the intervention time; 400 were found in PWS and 407 in SO. There were 399 common genes between the two groups. These genes were distributed in 17 VF classes (Supplemental Figure S1), among which the VF classes with a decreased proportion after dietary intervention in both PWS and SO groups included invasion (for efficient cellular invasion), T3SS (encoding type III secretory pathway proteins and effectors), exoenzyme, and toxin, whereas the VF classes with increased proportions included antiphagocytosis (to weaken host phagocytosis), iron uptake (for iron acquisition), and stress protein (for growth under stress conditions).

**Figure 1.**
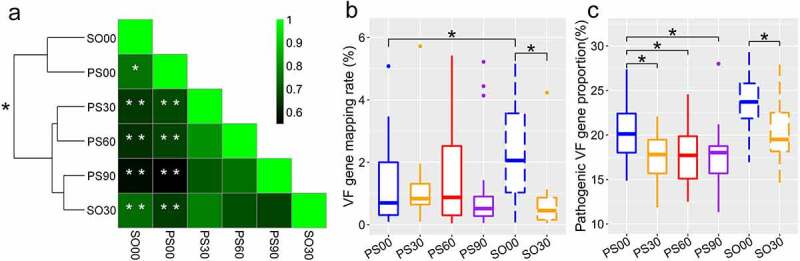
Dietary intervention alters gut microbial virulence factor (VF) genes in both Prader-Willi Syndrome (PWS) and simple obese (SO) groups. (a) The differences and correlations in the average abundance of VF genes between PWS and SO groups at different intervention time points. The groups were clustered with “Euclidean” and “Complete” methods based on the average abundance of the VF genes. The significance of the clustering is indicated with a black asterisk (PERMANOVA, * P < 0.05, permutations = 9,999). In the heatmap, the color indicates the Spearman correlation coefficient among groups, and the white asterisk indicates the significance of the ANOVA permutation test (* P < 0.05 and ** P < 0.01, permutations = 999). The mapping rate of the VF genes to the virulence factor database (VFDB) (b) and the relative proportion of pathogen-specific VF genes (c) of PWS and SO groups at different intervention time points. Boxes denote the medians and interquartile ranges (IQRs), and the whiskers denote the lowest and the highest values that were within 1.5 times the IQR from the first and third quartiles, respectively. A Wilcoxon matched-pair test (two-tailed) was used to analyze each pairwise comparison within groups. A Wilcoxon unpaired test was used to analyze differences between the PWS and SO cohorts before or after the dietary intervention. *P < 0.05 (adjusted by the Benjamini-Hochberg procedure). For PWS, n = 17 on day 0 (PS00), 30 (PS30), 60 (PS60), and 90 (PS90); For SO, n = 19 on day 0 (SO00) and day 30 (SO30)

The clustering results indicated that VF gene structures in both PWS and SO groups had significantly shifted based on the VF gene abundance (Equation 1; PERMANOVA test, permutations = 9,999, P < 0.05, Figure 1(a)). The ANOVA permutation test based on VF gene abundances showed that the VF gene composition of the PWS group was significantly different from that of the SO group before the dietary intervention (permutations = 999, PS00 vs. SO00, P < 0.05, Figure 1(a)). This variation declined at the end of the intervention, as PS90 was clustered with SO30 with no significant difference (PS90 vs. SO30, P > 0.05, Figure 1(a)).

Compared with the SO children, the PWS group had higher inflammation on day 0; thus, they had to undergo longer dietary intervention to fulfill the pre-defined clinical therapy endpoint. Surprisingly, the PWS group had a decreased total VF gene load on day 0 compared to that in the SO group (PS00 vs. SO00, P < 0.05, Figure 1(b)). The decrease in total VF gene load in PWS was not significant after the dietary intervention unlike that in the SO group (PS00 vs. PS30, PS60 or PS90, P > 0.05; S00 vs. SO30, P < 0.05, Figure 1(b)), which also suggested that the two cohorts displayed different responses in gut VFs to the same intervention. However, by classifying the detected VF genes into pathogen-specific and common VF genes [22], we found that the intervention effectively diminished the proportion of pathogen-specific VF genes in both cohorts (Figure 1(c)). Considering that both groups had improved clinical inflammation indexes, this result suggested that a decrease in the pathogen-specific VF genes instead of the total VF genes might be more relevant to the improvement of host inflammation.

### Shift in gut microbial VF genes in SO and PWS groups induced by dietary intervention

**Figure 2.**
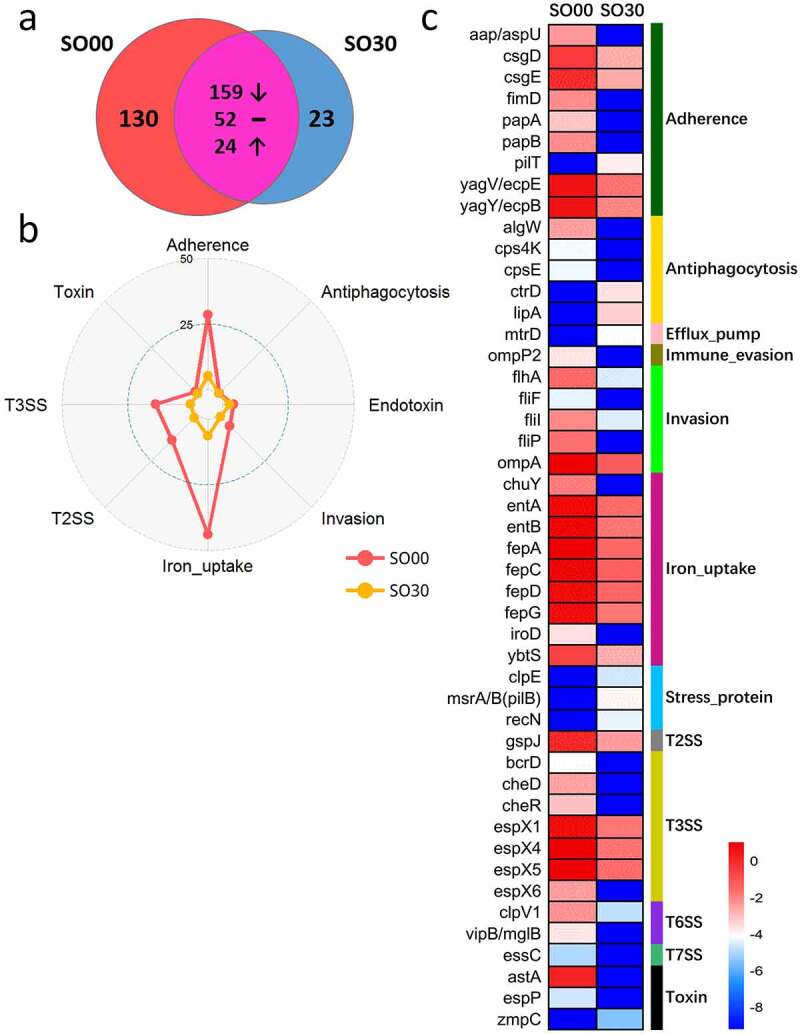
Changes in gut virulence factor (VF) genes after the intervention in the simple obese (SO) group. (a) The prevalence of VF genes in the SO group on dietary intervention day 0 (SO00) and 30 (SO30). (b) Radar chart shows the top eight abundant VF classes that decreased after the intervention. Each spoke in the chart represents one VF class, and the concentric circle indicates the abundance. (c) The relative abundance (natural log transformed) of the 47 featured differential VF genes before and after intervention in the SO group. These VF genes were selected using random forest feature selection and with a more than 8-fold change in abundance between SO00 and SO30

The SO group had an impressively decreased abundance of VF genes after the intervention. Of the 388 VF genes detected in the SO cohort, 130 were unique and present before the intervention (Figure 2(a)). These genes mainly encoded offensive and defensive VFs, belonging to the following classes: adherence, T3SS, invasion, antiphagocytosis, toxin, and exoenzyme. The 23 new VF genes that appeared after the intervention were less virulent than the VF genes present before the intervention, and they mainly encoded VFs belonging to adherence, antiphagocytosis, efflux pump (drug efflux pump), and stress protein (for growth under stress conditions) classes. There were 235 VF genes present throughout the intervention; the abundance of 159 genes (mainly encoding offensive and nonspecific category VFs) decreased, the abundance of 24 VF genes (encoding defensive and nonspecific category VFs) increased, and the abundance of the remaining 52 genes that encoded defensive and nonspecific category VFs remained unaffected (Figure 2(a)).

At the class level, the abundance of the top eight abundant VF genes, especially the genes encoding iron uptake, T3SS, T2SS, adherence, and invasion, declined after dietary intervention (Figure 2(b)). At the gene level, there were 47 remarkably changed featured differential VF genes after dietary intervention in the SO group, which were selected by random forest with more than an 8-fold change in abundance between before and after the intervention. Moreover, most of these genes decreased in abundance after the dietary intervention, such those related to adherence, invasion, iron uptake, T3SS, and toxin, whereas only the VF genes belonging to antiphagocytosis and stress protein increased in abundance after the intervention (Figure 2(c)). Among them, the invasion VF genes (*cheR, cheD, flhA, fliI3, fliP, fliF*, and *ompA*) mainly encoded flagella and outer membrane proteins and were derived from *Yersinia enterocolitica* subsp. *enterocolitica* 8081. The VF genes of the T3SS (*espX1, espX4, espX5, espX6*, and *bcrD*) from *Escherichia coli* O157:H7 str. EDL933 mainly encoded T3SS effectors. Moreover, the iron uptake genes (*fepC, fepG, entA, fepD, entB, fepA, iroD, chuY*, and *ybtS*) were mainly from *E. coli* CFT073 and encoded the ferrienterobactin ABC transporter.

**Figure 3.**
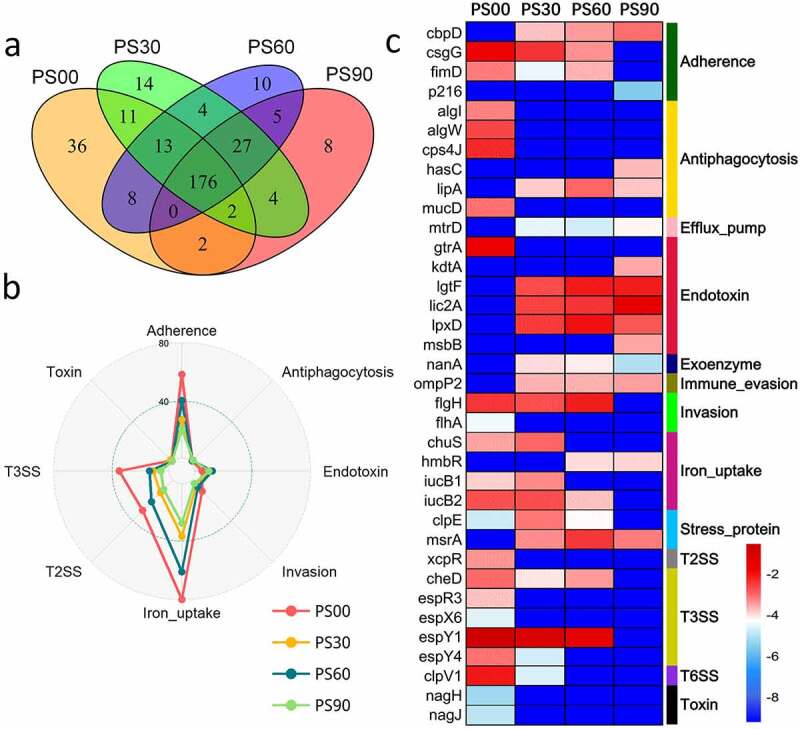
Changes of gut virulence factor (VF) genes along with the dietary intervention time in the Prader-Willi Syndrome (PWS) cohort. (a) The prevalence of the VF genes in the PWS cohort on dietary intervention day 0 (PS00), 30 (PS30), 60 (PS60), and 90 (PS90). (b) Radar chart shows that the top eight abundant VF classes decreased after the intervention. Each spoke in the chart represents one VF class and the concentric circle indicates the abundance. (c) The relative abundance (natural log transformed) of the 36 featured differential VF genes before and after the intervention in the PWS cohort. These VF genes were selected using random forest feature selection and with a more than 8-fold change in abundance between PS00 and PS90

In the PWS cohort, the diversity of the detected VF genes diminished as the intervention progressed (Figure 3(a)), as only 176 of the 320 VF genes remained. The diversity and abundance of the offensive and nonspecific VF genes decreased by the end of the dietary intervention. These genes mainly encoded VFs belonging to adherence, invasion, T3SS, T2SS, and iron uptake classes. However, the abundance of VF genes belonging to the endotoxin class increased after the dietary intervention (Figure 3(b) and Supplemental Figure S1). Since PWS-group children needed a longer intervention to reach clinical endpoints, PS90 was chosen as the representative time point for this cohort after the intervention. We identified 36 featured differential VF genes between PS00 and PS90; the abundance of genes encoding VFs belonging to adherence, antiphagocytosis, invasion, iron uptake, T3SS, and toxin classes decreased after the dietary intervention, whereas that of genes encoding VFs belonging to the endotoxin class increased (Figure 3(c)). We found that these endotoxin VF genes mainly encoded LOS (lipooligosaccharide) of *Haemophilus* according to VFDB, and the abundance of *Haemophilus* increased after dietary intervention in the PWS group [24] (Supplemental Figure S2(a)). The VF genes of T3SS (*espX6, espY4, espY6, espR3* and *cheD*) were mainly from *E. coli* O157:H7 str. EDL933 and encoded T3SS effectors, similarly to that in SO children. The toxin VF genes (*nagH* and *nagJ*) from *Clostridium perfringens* str. 13 encode hyaluronidase, which can degrade the connective tissue during gas gangrene [41].

### Dietary intervention narrows the structural difference in VF genes between PWS and SO groups

**Figure 4.**
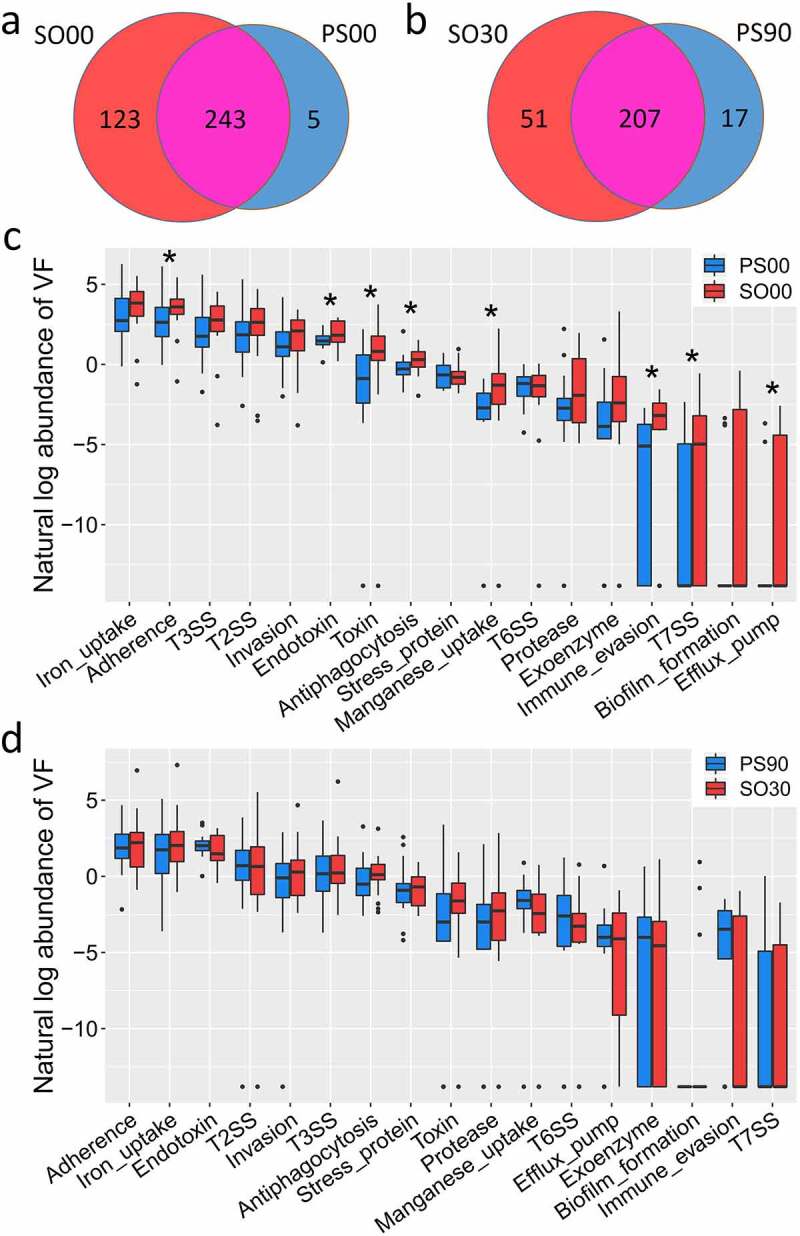
Comparison of the detected virulence factor (VF) genes between Prader-Willi Syndrome (PWS) and simple obese (SO) cohorts. (a) The prevalence of the gut VF genes between PWS (PS00) and SO (SO00) before the intervention. (b) The prevalence of the gut VF genes between PWS (PS90) and SO (SO30) after the intervention. The abundance (natural log transformed) of VF gene classes in the PWS and SO groups before (c) and after (d) the intervention. Boxes denote the medians and interquartile ranges (IQRs), and the whiskers denote the lowest and the highest values that were within 1.5 times the IQR from the first and third quartiles, respectively. A Wilcoxon test (two-tailed) was used to analyze differences between the PWS and SO groups. *P < 0.05 (adjusted by the Benjamini-Hochberg procedure)

The initial VF genes carried by the SO and PWS groups were different. Though 243 VF genes were detected in both cohorts, the SO cohort had more unique VF genes (123) than the PWS cohort (five genes) before the dietary intervention (Figure 4(a)). Compared with that in the PWS cohort, the SO cohort not only had a higher Shannon-Wiener diversity index but also an increased abundance of offensive and defensive VF genes, particularly belonging to adherence, endotoxin, antiphagocytosis, and toxin classes (Figure 4(c) and Supplemental Figure S2(b)).

**Figure 5.**
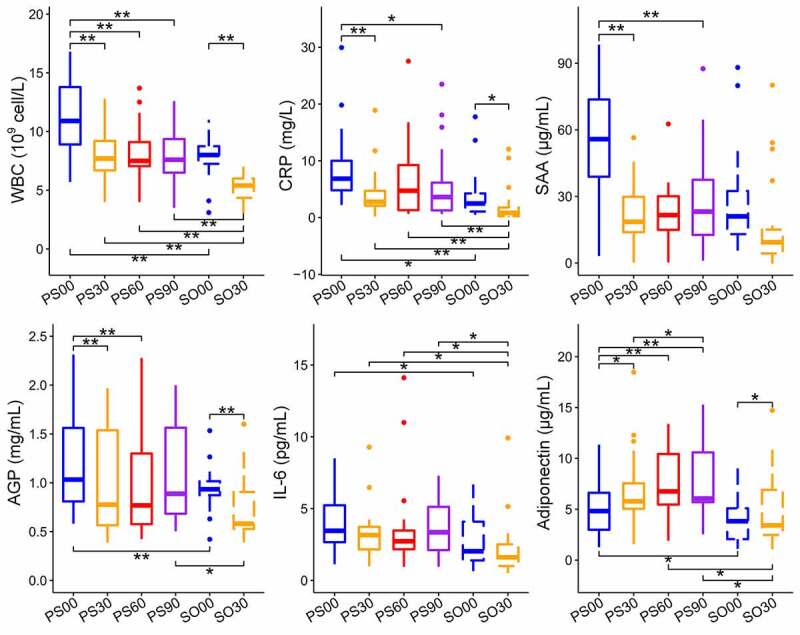
Dietary intervention decreases the inflammation indexes in Prader-Willi Syndrome (PWS) and simple obese (SO) groups. Changes in the six inflammation indexes (WBC, CRP, SAA, AGP, IL-6, and adiponectin) in PWS and SO groups based on different dietary intervention days were determined. Boxes denote the medians and the interquartile ranges (IQRs), and the whiskers denote the lowest and highest values that were within 1.5 times the IQR from the first and third quartiles, respectively. A Wilcoxon matched-pair test (two tailed) was used to analyze each pairwise comparison within groups. A Wilcoxon unpaired test was used to analyze differences between the PWS and SO groups before or after the dietary intervention. *P < 0.05, **P < 0.01 (adjusted by the Benjamini-Hochberg procedure). WBC: white blood cell count; CRP: C reactive protein; SAA: serum amyloid A protein; AGP: α-acid glycoprotein; IL-6: interleukin 6

The difference in VF genes was reduced between the two cohorts at the end of the clinical trial. The number of unique VF genes in the SO cohort was reduced from 123 to 51 after the dietary intervention; however, this number was larger than that in the PWS cohort (51 vs. 17). The two cohorts had 207 VF genes in common, and there was no considerable difference in VF class abundance (Figure 4(b, d)). Considering the fact that the PWS cohort had a higher inflammation level but with a decreased abundance of VF genes compared to those in the SO group (Figure 5), VF structure rather than load might be responsible for host inflammation.

### Clustered VF genes related to inflammation in the PWS cohort

**Figure 6.**
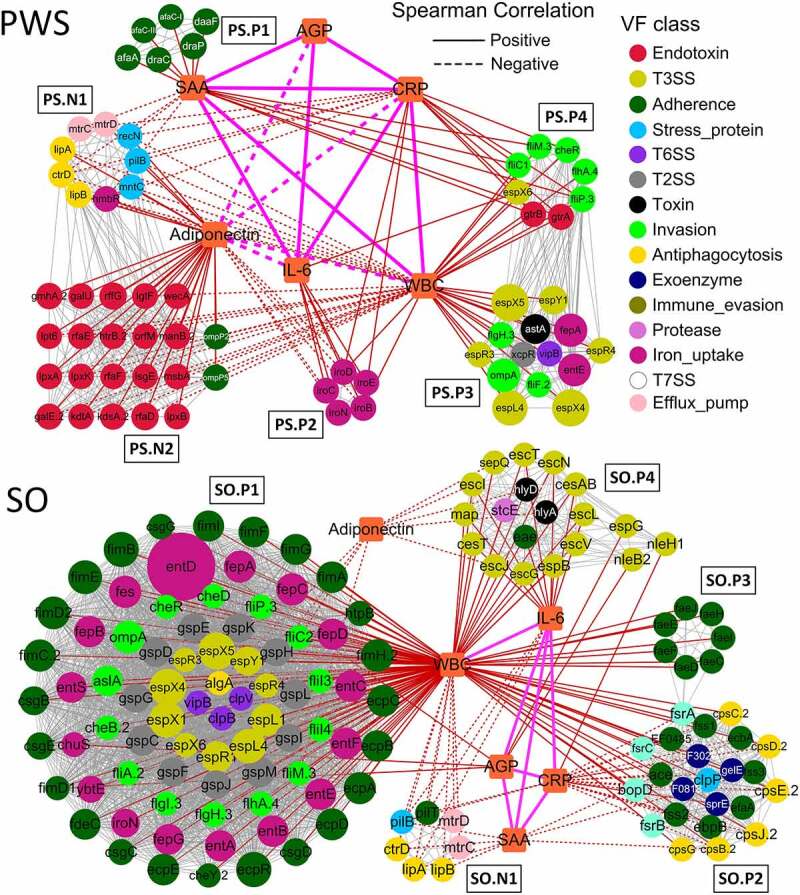
Clustered virulence factor (VF) genes related to the inflammation indexes are different between Prader-Willi Syndrome (PWS) and simple obese (SO) cohorts. The co-occurrence network of VF genes and inflammation indexes (WBC, IL-6, CRP, SAA, AGP, and adiponectin) in PWS and SO cohorts is shown. Each node represents one VF gene, and the color and size of the node indicate the class and the abundance of the VF genes, respectively. Squares represent the inflammation indexes. Red lines represent Spearman correlations between the VF genes and the inflammation indexes; gray lines represent the correlations between the VF genes; orange lines represent the correlations between the indexes. WBC: white blood cell count; CRP: C reactive protein; SAA: serum amyloid A protein; AGP: α-acid glycoprotein; IL-6: interleukin 6

To explore the relationship between the VF genes and inflammation in obese children, we constructed a co-occurrence network of VF genes and the six inflammation indexes based on the Spearman correlations. In the PWS cohort network, we found that four VF clusters (PS.P1, PS.P2, PS.P3, and PS.P4) and two VF clusters (PS.N1 and PS.N2) were positively and negatively related to the inflammation indexes, respectively (Figure 6). There were six VF genes in cluster PS.P1 that were positively related to SAA. SAA is synthesized in hepatocytes and inflammatory tissue by macrophages, monocytes, and endothelial cells; it has a close association with cholesterol and platelets and controls cholesterol transport and clearance from inflammatory sites [42,43]. The VF genes (*draC, draP, afaA, afaC-I, afaC-III*, and *daaF*) in cluster PS.P1 encoded Afa/Dr adhesins from *E. coli*, which are widespread systems for the secretion of fimbrial proteins, display an afimbrial/fimbrial morphology, and are correlated with diffused adherence, invasion, and increased expression of vascular endothelial growth factor in intestinal epithelial cells [44]. Cholesterol-dependent lipid rafts play a role in Afa/Dr pathogenicity, along with the glycosylphosphatidylinositol (GPI) anchored proteins CD55 and carcinoembryonic antigen (CEA) as receptors [45,46], and SAA is closely associated with cholesterol transport [47].

The five clustered VF genes (*iroB, iroC, iroD, iroE*, and *iroN*) from *E. coli* CFT073 in PS.P2 were positively related to WBC, CRP, and IL-6 and negatively related to adiponectin. WBC of the immune system circulates in the blood and is involved in phagocytosis and the clearance of immune complexes and apoptotic cells, among other functions [48]. CRP plays a role in innate immunity by binding to phosphocholine and promoting the phagocytosis of necrotic cells and bacteria by macrophages [49]. IL-6 is a pleiotropic cytokine required for the hepatic production of APPs and participates in the acquisition of immunity against chronic intracellular infections [50,51]. Adiponectin shows potent anti-inflammatory properties by reducing macrophage differentiation and migration, producing chemokines, and suppressing T-cell migration [52]. The genes related to these four indexes in PS.P2 were found to be involved in iron uptake in uropathogenic *E. coli* CFT073. The VF genes *iroBCDEN* encode the salmochelin siderophore system and play important roles in the acquisition of iron (Fe3+) and virulence during infection [53]. The VF genes in cluster PS.P3 were positively related to WBC and mainly encoded T3SS effector (*espL4, espR3, espR4, espX4, espX5*, and *espY1*), flagellar (*flgH* and *fliF*), outer membrane (*ompA*), and heat-stable enterotoxin (*astA*), which were associated with strong infectivity [54,55]. The clustered VF genes in PS.P4 encoded flagellar (*fliC1, fliM, fliP, flhA* and *cheR*), LPS (*gtrA* and *gtrB*), and T3SS effector (*espX6*), were positively related to WBC, CRP, and SAA, and were mainly from *Y. enterocolitica* subsp. *enterocolitica* 8081 and *Shigella flexneri* 2a str. 301.

Cluster PS.N1 was negatively related to WBC, SAA, and CRP and positively related to adiponectin. The VF genes (*ctrD, mtrC, mtrD, pilB, hmbR, mntC*, and *recN*) in PS.N1 were similar to those from *Neisseria meningitidis* MC58 (source) and were associated with stress proteins (quenching ROS and repairing oxidized proteins), capsule formation (protecting against phagocytosis, antimicrobial peptides, and killing by complement), and efflux pumps (protecting bacteria from antibiotics and antimicrobial peptides) [56]. Cluster PS.N2 was composed of 20 endotoxin (LOS) and two outer membrane protein encoding VF genes (*ompP2* and *ompP5*), which were negatively related to WBC, IL-6, and CRP and positively related to adiponectin. All VF genes in PS.N2 were similar to those reported from *Haemophilus influenzae* Rd KW20 (source), as a nontypeable *H. infuenzae* (NTHi), which is etiologically associated with a range of acute and chronic diseases [57]. LOS of *H. influenza* affects the ability to adhere to and invade cells effectively and has a critical role in the induction of inflammation and resistance to complement [58,59]. The two outer membrane proteins, OMP-2 and OMP-5, bind to host cells to release TNF-α and IL-6 [60] and trigger the expression of CD105 [61], respectively. AGP can bind and carry hydrophobic molecules, which is a consequence of the inflammation process, including the inhibition of neutrophil activation and increases in the secretion of the IL-1 R antagonist by macrophages [43,62]. AGP did not correlate to clustered VF genes but were positively related to three inflammation indexes, namely SAA, CRP, and IL-6, in the PWS group.

### Clustered VF genes related to inflammation in the SO cohort

For the SO cohort network, five crowded VF clusters, namely SO.P1, SO.P2, SO.P3, SO.P4, and SO.N1, were closely related to the inflammation indexes (Figure 6). The VF genes in cluster SO.P1 were abundant and positively related to WBC. These VF genes belonged to the following classes: adherence, iron uptake, invasion, and secretion systems (T3SS, T2SS, and T6SS). Adherence VF genes in SO.P1 encoded three products, *E. coli* common pilus (ECP), curli, and type 1 fimbriae. The VF genes encoding ECP (*ecpA, ecpB, ecpC, ecpD, ecpE*, and *ecpR*) were from *E. coli* O157:H7 str. EDL933. As an accessory adherence factor, ECP is necessary to promote cell adherence in bacterial interactions and plays a dual role in biofilm development and host cell recognition [63,64]. The VF genes encoding curli (*csgB, csgC, csgD, csgE*, and *csgG*) were from *Salmonella enterica* subsp. *enterica* Serovar Typhimurium str. LT2. Curli, a pathogen associated molecular pattern (PAMP), interacts with molecules of the host immune system, which is important for surface colonization, by enhancing adherence and inducing a proinflammatory response [65]. The VF genes encoding type 1 fimbriae (*fimA, fimB, fimC.2, fimD2, fimE, fimF, fimG, fimH.2*, and *fimI*) were from *E. coli* CFT073. Type 1 fimbriae as PAMPs provide Toll-like receptor (TLR) 4 and LPS-dependent signals to CD14-negative epithelial cells and induce cytokine production [66,67].

Sixteen iron uptake VF genes in SO.P1 encoded enterobactin similar to that isolated from uropathogenic *E. coli* CFT073 (source). Enterobactin is a powerful ferric chelator, which recognizes and transports ironbound salmochelin to keep iron stores secure during competition with the host [68]. A VF gene called *entD* with relatively high abundance encoding an extremely effective iron chelator was also detected. Invasion VF genes in SO.P1 mainly encoded proteins associated with flagella and chemotaxis of the highly pathogenic bacterium *Y. enterocolitica* subsp. *enterocolitica* 8081. As reported, flagella and chemotaxis play roles in moving toward favorable conditions or avoiding stressful environments, and these proteins can also be recognized by Toll-like innate immune receptors of the host [69,70]. There were 24 secretion system VF genes (10 T3SS, 11 T2SS, and three T6SS) in SO.P1. Among them, the VF genes encoding T3SS effectors (*espL1, espR1* and *espX1*) were derived from *E. coli* O157:H7 str. EDL933. Virulence effector proteins encoded by the T3SS VF genes can be injected into the host cells; they can modify signal transduction pathways, interfere in phagocytosis by host cells, and suppress innate immunity [54,55]. General secretion pathway (GSP) proteins encoded by the T2SS genes deliver toxins, pilus proteins, curli, invasins, adhesins, and proteases to the extracellular environment [71,72]. These VF genes encoding GSP proteins (*gspC, gspD, gspE, gspF, gspG, gspH, gspI, gspJ, gspK, gspL*, and *gspM*) were from *Shigella dysenteriae* Sd197. The T6SS VF genes (*clpB* and *clpV*) were related to the ATPase and tubule-forming protein, which are responsible for delivering protein effectors into the host cell via a contractile ejection apparatus [73,74].

Clustered VF genes in SO.P2 were mainly from the following classes: adherence, antiphagocytosis, exoenzyme, and biofilm formation, which were positively related to WBC but negatively related to SAA and CRP. The abundance of these genes declined after dietary intervention (Supplemental Figure S3). Eight adherence VF genes (*ace, ebpB, ecbA, efaA, fss1, fss2, fss3*, and *EF0485*) from *Enterococcus faecalis* V583 in SO.P2 were determined to be involved in binding to fibrinogen, collagen, and fibronectin [75]. Six VF genes (*cpsB, cpsC, cpsD, cpsE, cpsG*, and *cpsJ*) in SO.P2, encoding the antiphagocytic capsule of *E. faecalis* V583, contribute to escape detection and clearance by the host immune system and are widely distributed within species due to horizontal gene transfer [76,77]. Additionally, there were four exoenzyme genes (*EF0818, EF3023, gelE*, and *sprE*) and four biofilm formation genes (*fsrA, fsrB, fsrC*, and *bopD*) from *E. faecalis* V583 in SO.P2. The exoenzyme VF genes encode hyaluronidase, gelatinase, and serine proteinase, which enable colonization and invasion of host tissues and provide nutrients to the bacteria by degrading host tissue [74]. Moreover, the exoenzyme VF genes also have some functions in biofilm formation. Genes *sprE* (encoding serine protease) and *gelE* (encoding gelatinase) are co-transcribed and jointly regulated by quorum-sensing system proteins that are encoded by the *fsr* (fecal streptococci regulator) locus [78]. SO.P3 was only positively related to WBC, which contained seven clustered adherence VF genes (*faeC, faeD, faeE, faeF, faeH, faeI*, and *faeJ*) from *E. coli* SE11. These genes encode F4 (K88) fimbriae that are composed of long filamentous polymeric surface proteins and could mediate binding to F4 specific receptors on brush borders of villous enterocytes to colonize the small intestine [79,80].

SO.P4 mainly consisted of 15 T3SS and two toxin VF genes. This cluster was positively related to WBC and IL-6 and negatively related to adiponectin. Most of the T3SS VF genes from *E. coli* O157:H7 str. EDL933 encode the structural components of the T3SS, for example, the inner rod component, ring protein, and export apparatus protein. Meanwhile, there were three other VF genes (*nleH1, nleB2*, and *espG*) that encoded T3SS effector proteins and were positively related to AGP and CRP, which interfere with the activation of NF-κB and affect the tight junction function of human epithelial cells [81,82]. Additionally, the two toxin VF genes (*hlyA* and *hlyD*) from *E. coli* CFT073, encoding hemolysin, and one protease gene, *stcE*, encoding metalloprotease, could cause proinflammatory responses and result in tissue damage during infection [83,84]. Moreover, cluster SO.N1 was negatively related to WBC, IL-6, and SAA and was composed of three antiphagocytosis genes (*ctrD, lipA*, and *lipB*), two efflux pump genes (*mtrC* and *mtrD*), one stress protein gene (*pilB*), and one adherence gene (*pilT*). Similar to PS.N1 VF genes, the VF genes in SO.N1 were from *N. meningitidis* MC58 and involved in quenching ROS, repairing oxidized proteins, and protecting against phagocytosis and antimicrobial peptides, among others [56].

All VF genes that appeared in the co-occurrence networks along with their annotation information are listed in Supplemental Table S2. In SO, there were 128 and seven VF genes positively and negatively related to inflammation, respectively. In contrast, in PWS, there were 33 and 32 VF genes that were positively and negatively related to inflammation, respectively. The positive VF genes that were common to the PWS and SO networks mainly belonged to invasion (*cheR, flgH, flhA, fliM, fliP*, and *ompA*) and T3SS (*espL, espX, espY*, and *espR*) classes, implying that they might play an important role in promoting the occurrence and development of inflammation in both PWS and SO groups. These positive VF genes were from *E. coli* O157:H7, *Y. enterocolitica subsp. enterocolitica* 8081, and *E. coli* CFT073, whereas the negative VF genes were mainly from *N. meningitidis* MC58.

## Discussion

Our previous clinical trial elucidated that a high-fiber dietary intervention could suppress inflammation in obese children, which could be due to modulation of the gut microbiota structure. The investigation on VF genes performed in the current study suggests that our dietary intervention improved the inflammation status of obese children through reductions in infective and reproductive VF genes and changes in VF gene structure from more invasive to less invasive, thus weakening the overall damaging effect of VFs.

Gut pathogen-specific VF genes decreased in both PWS and SO cohorts after the dietary intervention. These decreased VF genes were pathogen-specific and were responsible for damage to host cells, such as, colonization, invasion, and inflammation. The reduction in gut pathogen-specific VF genes was rapid, as a major change in their abundance was observed on day 30 (the first sampling point after the intervention). Moreover, the inflammatory conditions in both types of obese children were also alleviated, implying an association between the gut VF genes and host inflammation. Additionally, the impressive differences in VF genes present in PWS and SO cohorts before the intervention were diminished at the end of the clinical trial, indicating a convergent and beneficial effect of the high-fiber diet for obese children.

Different responses of VF genes to the dietary intervention were also identified between the PWS- and SO-group children. First, increased abundance of VF genes was detected in SO children compared to that in the PWS group, and the response of VF genes to the high-fiber dietary intervention was more rapid in the SO group than in the PWS group. Second, the functions and products of the VF genes closely related to inflammation indexes were inconsistent between PWS and SO groups, although the three major VF classes in both cohorts were iron uptake, T3SS, and invasion. The VF genes of iron uptake related to inflammation were responsible for the salmochelin siderophore system in the PWS cohort but for enterobactin in the SO cohort. The VF genes of the T3SS only encoded T3SS effectors in the PWS cohort, whereas these T3SS genes also encoded structural components of the T3SS in the SO cohort. The invasion VF genes relevant to host inflammation in the SO cohort were responsible for flagella and chemotaxis, but in the PWS cohort, they were only responsible for the synthesis of flagella. Additionally, the VF genes in the SO cohort encoded GSP protein, type 1 fimbriae, faecalis surface protein, ECP, K88 pili/F4 fimbriae, and antiphagocytic capsule, but these functions were not observed in the PWS cohort. This indicated that the dietary intervention improved the inflammation condition of PWS and SO groups by altering the VF structures via different mechanisms.

The PWS group had a lower abundance of VF genes and detrimental VF clusters related to host inflammation but displayed more inflammation than the SO children group, both before and after the dietary intervention. Several other studies have also reported that PWS patients have increased low-grade inflammation compared to the matched SO patients [85–87]. Viardot and Caixàs found that the PWS group not only had more inflammation but also showed more activated innate immunity than SO and healthy subjects [86,87]. Taken together, we suspected that the activated innate immune system associated with PWS might have contributed to the aggravation of inflammation.

Increased abundance of endotoxin (LOS) VF genes derived from *H. influenzae* Rd KW20 might also account for higher inflammation in the PWS group than in the SO group. Coincidentally, Qin et al found that *Haemophilus* was considerably enriched in control individuals relative to levels in T2D patients, which could be beneficial for the host under these circumstances [88]. Qin et al identified a decline in *Haemophilus* in H7N9-infected patients compared to levels in healthy controls and implied an unknown function for this pathogen [89]. Alternatively, in this study, the abundance of *Haemophilus* was lower in obese children with higher inflammation than in healthier individuals, implying that *Haemophilus* might be subjected to competitive antagonism from other functionally dominant pathogens that carry toxic or invasive VFs and survive under these hostile circumstances (i.e., more inflammatory and obese host conditions). Further studies are required to understand this phenomenon. However, considering that LOS could be recognized by TLRs and activate the inflammatory response [58,59], the increase in the abundance of LOS VF genes and their carrier *Haemophilus* could be one way to explain why the PWS group had higher inflammation than the SO group after dietary intervention.

Another interesting finding was that the VF genes involved in quenching ROS and repairing oxidized proteins from *N. meningitidis* were increased after the high-fiber dietary intervention in both PWS and SO groups. Some studies have shown an increase in the generation of ROS and oxidative stress from accumulated adipocytes in obesity [90,91]. Uberos found that obese children carried more *N. meningitidis* (in their respiratory tract) than non-obese children, and the plasma antioxidant capacity decreased in asymptomatic children carrying *N. meningitidis*; they thus hypothesized that the children carrying *N. meningitidis* had an oxidative balance favorable to the compensatory consumption of antioxidant molecules [92,93]. Several case-control studies based on the gut metagenome also showed that *N. meningitidis*, as an opportunistic pathogen, is widely present in the gut of healthy and diseased individuals [94,95]. Therefore, we speculated that even though the obese children had reduced body weight and inflammation after the dietary intervention, they were still categorized in the obese range and consequently faced cumulative oxidative stress; thus, *N. meningitidis* could have taken advantage of its ability to quench ROS during competition with other gut bacteria. Hence, dietary interventions should be continued for longer periods to achieve better health effects for obese children.

In this study, the detection and analysis of gut microbial VF genes revealed that some opportunistic pathogens were prevalent in obese children, such as *N. meningitidis, Y. enterocolitica*, and *H. influenza*, although their abundance was relatively low. Those results led us to consider the hypothesis that some opportunistic pathogens could exist in the intestinal environment of special cohorts due to their specific characteristics, such as quenching ROS. Further, more studies on gut microbial VFs in healthy and diseased individuals are needed to confirm the prevalence of opportunistic pathogens in the host gut.

The time series metagenomic data in this study allowed us to perform systematic investigations on multiple VF genes, to observe the influence of a high-fiber diet, and to understand their potential roles in the regulation of inflammation in PWS- and SO-group children. In this study, we used an effective strategy to study the relationship between gut VF genes and host health indicators and found that the dietary intervention decreased pathogenic VF genes, shifted the VF gene structure, and improved inflammation conditions in the PWS and SO groups by altering the functions and classes of VF genes. Those phenomena led us to presume that the shifted gut microbial VFs, mediated by a high-fiber diet, might lead to the changes in the host inflammation response. However, given the relatively small sample size for clinical gut microbiota research, further validation of the results with a larger sample size is required. The results obtained by this investigation provide new insight into the developmental process of therapies to alleviate obesity and inflammation by targeting gut microbial VF genes.

## Supplementary Material

Supplemental Material
