# Supplementary figures and images for: Suppressed inflammation in obese children induced by a high-fiber diet is associated with the attenuation of gut microbial virulence factor genes
Source: Virulence. 2021 Jul 8;12(1):1754–70. doi: 10.1080/21505594.2021.1948252 (PMC8274444; doi:10.1080/21505594.2021.1948252)

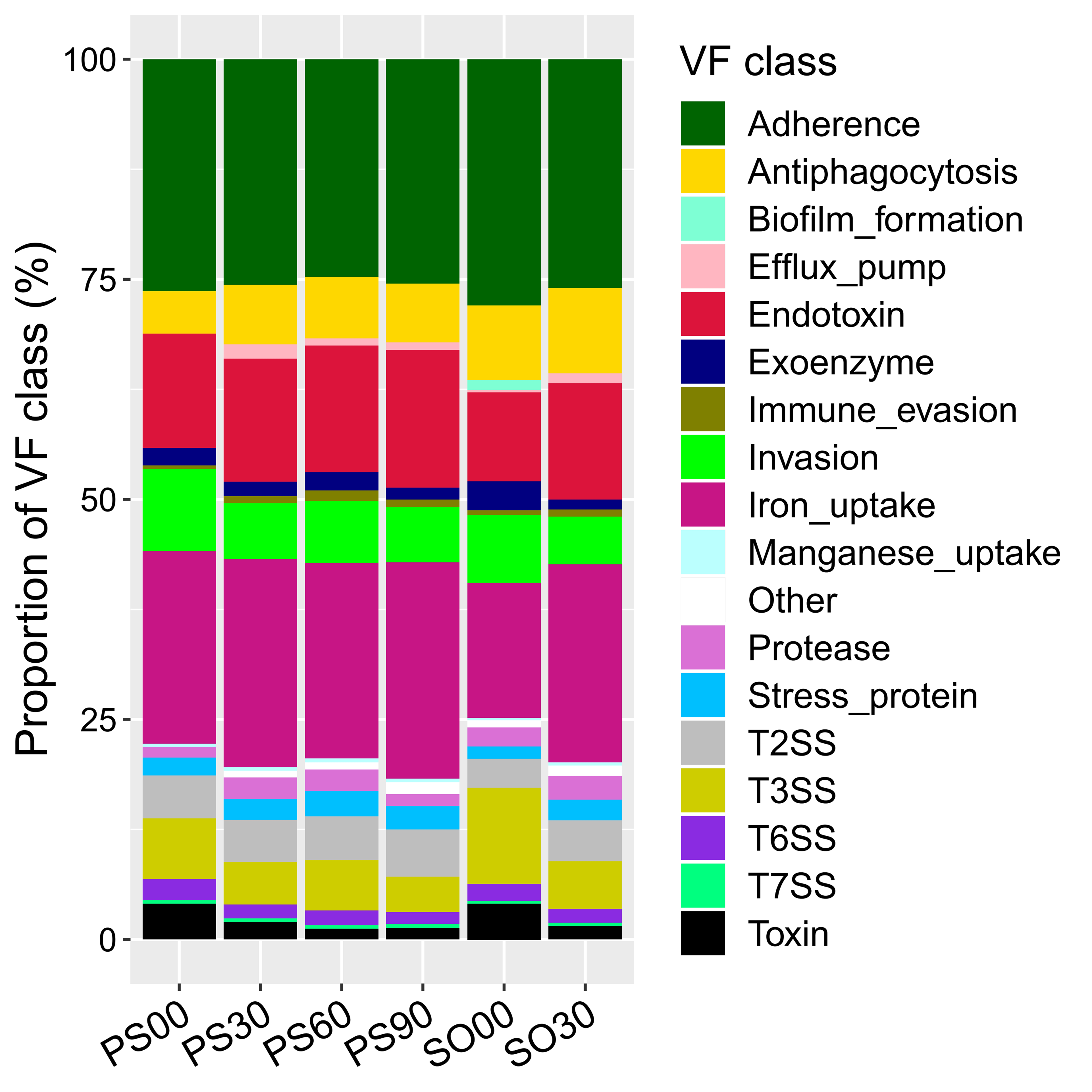

Supplement: Supplemental Material [file KVIR_A_1948252_SM5856.zip › supplementary/FigureS1.jpg]

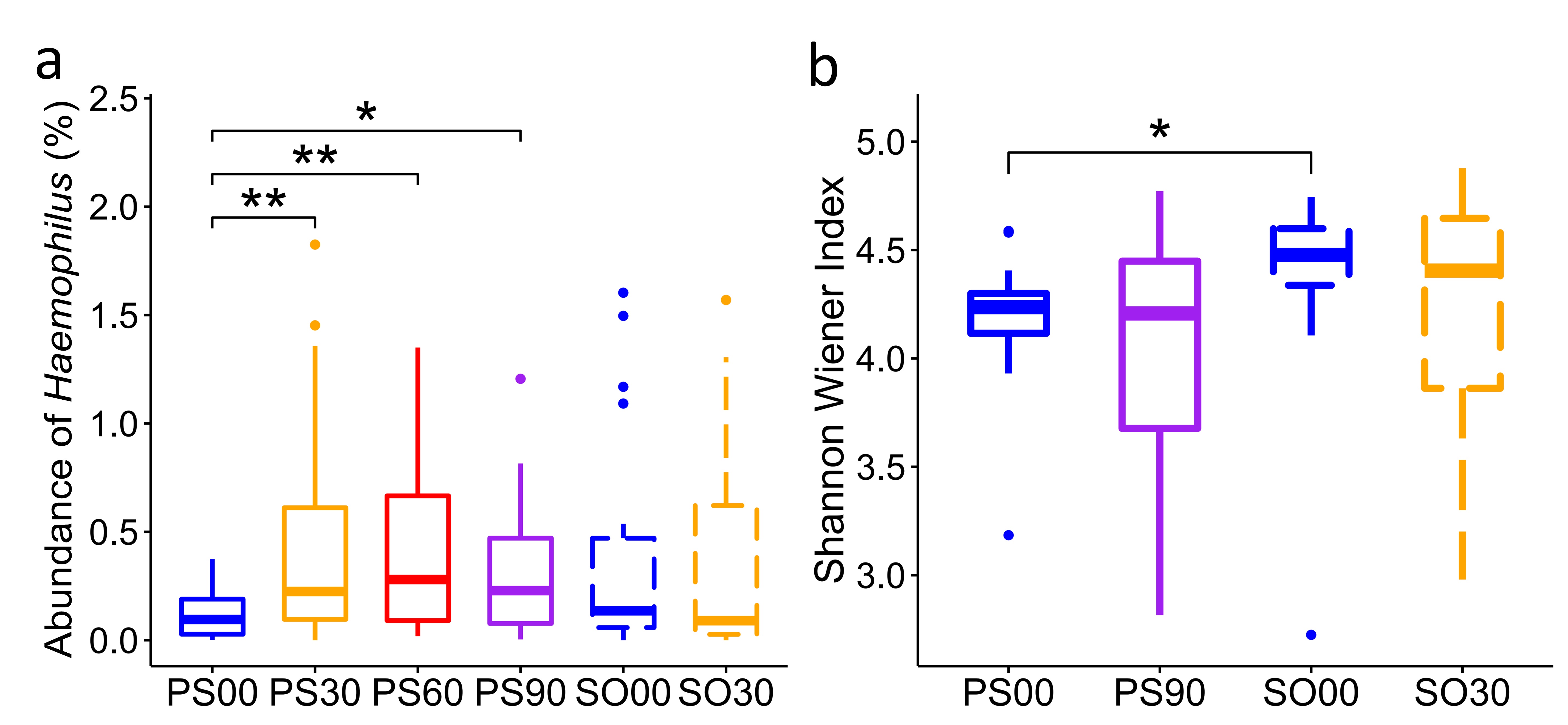

Supplement: Supplemental Material [file KVIR_A_1948252_SM5856.zip › supplementary/FigureS2.jpg]

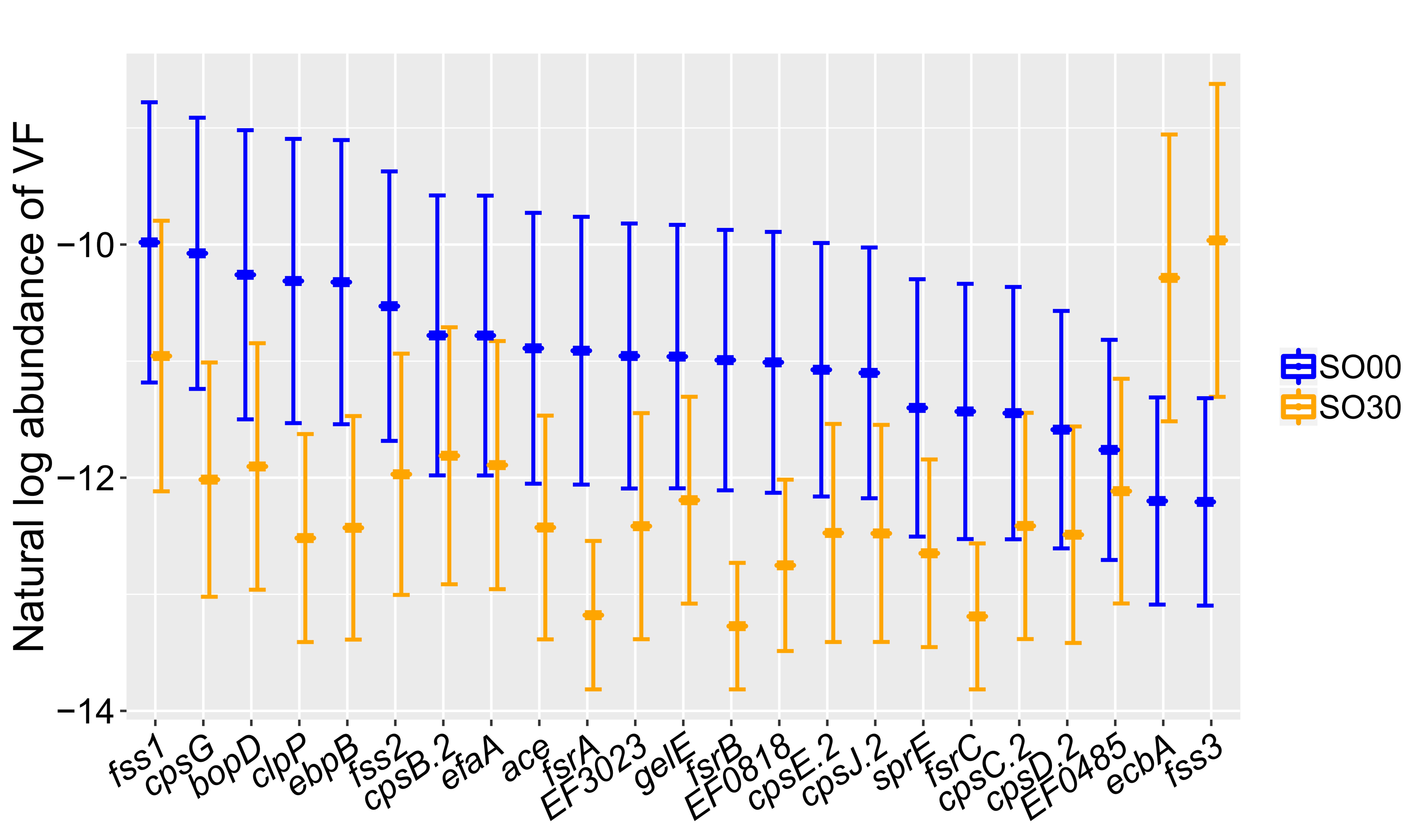

Supplement: Supplemental Material [file KVIR_A_1948252_SM5856.zip › supplementary/FigureS3.jpg]
